# Achieving hip fracture surgery within 36 hours: an investigation of risk factors to surgical delay and recommendations for practice

**DOI:** 10.1007/s10195-015-0387-2

**Published:** 2015-11-26

**Authors:** Adeel Aqil, Fahad Hossain, Hassaan Sheikh, Joseph Aderinto, George Whitwell, Harish Kapoor

**Affiliations:** Level 1 Trauma Centre, Leeds General Infirmary, Great George St, Leeds, LS1 3EX UK

**Keywords:** Hip fractures, Time to treatment, 36 h

## Abstract

**Background:**

The UK hip fracture best practice tariff (BPT) aims to deliver hip fracture surgery within 36 h of admission. Ensuring that delays are reserved for conditions which compromise survival, but are responsive to medical optimisation, would help to achieve this target. We aimed to identify medical risk factors of surgical delay, and assess their impact on mortality.

**Materials and methods:**

Prospectively collected patient data was obtained from the National Hip Fracture Database (NHFD). Medical determinants of surgical delay were identified and analysed using a multivariate regression analysis. The mortality risk associated with each factor contributing to surgical delay was then calculated.

**Results:**

A total 1361 patients underwent hip fracture surgery, of which 537 patients (39.5 %) received surgery within 36 h of admission. Following multivariate analyses, only hyponatraemia was deduced to be a significant risk factor for delay RR = 1.24 (95 % CI 1.06–1.44). However, following a validated propensity score matching process, a Pearson chi-square test failed to demonstrate a statistical difference in mortality incidence between the hypo- and normonatraemic patients [*χ*^*2*^(1, *N* = 512) = 0.10, *p* = 0.757].

**Conclusions:**

Hip fracture surgery should not be delayed in the presence of non-severe and isolated hyponatraemia. Instead, surgical delay may only be warranted in the presence of medical conditions which contribute to mortality and are optimisable.

**Level of evidence:**

III

## Introduction

A fracture of the hip is the commonest cause of injury-related death in the UK [1]. Prompt surgery has been associated with higher rates of independent living and lower 30-day and 1-year mortality rates [2–5]. Earlier surgery has also been shown to improve patient outcomes by reducing pain scores, and lowering of the risk of decubitus ulcer formation and length of inpatient stay [2, 6, 7].

The inception of best practice tariffs (BPTs), which aimed to improve these patient outcomes, stemmed from the ‘Equity and Excellence: liberating the NHS’ government white paper [8]. BPTs are incentivised targets, which financially compensate organisations for delivering high quality care. In the context of hip fracture management, the BPT consists of an initial base tariff, with additional payments if further criteria of best practice have been met. One of these criteria is delivering hip fracture surgery within 36 h of presentation to a health care institution. This government target is also in accordance with clinical guidelines set by the British Orthopaedic Association (BOA) and National Institute of Clinical Excellence (NICE), which state that hip fracture surgery should be performed on the day of, or the day after admission and within normal working hours [9, 10]. However, the National Hip Fracture Database (NHFD) has reported that this specific BPT target was met in only 71.4 % of hip fracture patients, equating to £15.9 million in ‘lost’ monetary incentives [1].

Clearly, resources must be made available to allow such a level of service provision and to qualify for the maximum financial reward the BPT has to offer. Optimal clinical decision-making could therefore augment and streamline management in order to facilitate early surgery. As survival is perhaps the most desirable outcome following a fracture of the neck of femur (FNOF), and delay to surgery in itself carries an increased risk to mortality, then it certainly follows that delays for medical optimisation would only be justified for conditions which also carry a mortality risk [3–7]. Therefore, identifying medical risk factors for surgical delay and their associated mortality risk would assist organisations to rationalise clinical decision-making, and thus enhance compliance with the BPT target.

The primary aim of this study was therefore to identify medical conditions associated with patients failing to achieve the 36-h cut-off for surgery following a hip fracture. We subsequently evaluated whether these factors were justifiable in risking surgical delay by gauging whether they were also associated with an increased risk to mortality.

## Materials and methods

We obtained prospectively collected hip fracture patient information from the UK NHFD from before April 2010 and prior to the inception of the 36-h BPT guideline. Data was subsequently cross-referenced with our institution’s patient records. The use of data after the introduction of the BPT guidelines may have risked missing patients with legitimate causes of delay, who may have had their surgery expedited to meet the 36-h target. Hence analysis of delays was performed on data pre-dating the BPT introduction, allowing all medical causes of delays to be identified and an assessment of their risk to mortality to be performed.

We collected patient-level information including demographic data, American Society of Anesthesiologists (ASA) grade at the time of surgery, fracture type, source of admission and walking ability [11]. All patient co-morbidity data was identified using the International Classification of Disease 10th revision (ICD-10) codes, and these were used to calculate the Charlson co-morbidity index for each patient as a separate variable [12]. Biochemical parameters collected included admission haemoglobin levels (Hb), white cell count (WCC), coagulation profile, urea and electrolyte levels. Time to surgery from presentation was also collected.

The primary outcome of interest was a delay to surgery over 36 h from initial hospital presentation. The secondary outcome examined was the occurrence and causes of mortality within 30 days of admission. Primary and secondary causes of death were noted from death certificates and hospital death records. A total of 1674 patients were initially identified, but following exclusions of incomplete data sets and incorrect or duplicate entries, a total of 1361 patients were included in the study.

Statistical analysis was undertaken in a two-stage process. We initially categorised patients into two groups: group1 = time to surgery <36 h; group 2 = time to surgery >36 h). All variables collected were then compared between these two groups on initial univariate analysis using the chi-square or Fisher’s exact test for categorical data and the independent *t*/Mann–Whitney test for continuous variables. A subsequent backward stepwise Cox regression model was undertaken to identify the most significant determinants of surgical delay beyond 36 h. Our criteria for inclusion of variables into the model included a *p* value <0.15 on univariate analysis, in accordance with published statistical methods [13]. Results were displayed as relative risks rather than odds ratios, in order to aid clinical interpretation [14].

The decision to delay hip fracture surgery on medical grounds is undertaken to avoid significant complications which may result from precipitous surgery. Death is perhaps the most important complication to avoid. Therefore, it is logical to validate variables that risk a delay to surgery beyond 36 h in terms of their impact on mortality. We undertook a second-stage analysis to assess mortality likelihood at 30 days following surgery for each individual variable which had been found to delay surgery. To limit potential for selection bias, when assessing one variable’s association with mortality we had to control for all other variables. We therefore derived a single scalar propensity score from the regression of all remaining covariates in Tables 1 and 2. Between-group propensity score matching was performed using a “nearest neighbour” matching strategy [15]. An assessment of the matching process consisted of an evaluation of between-group standardised mean differences and variance ratios according to published standards [16]. Between-group mortality analysis used a chi-square test for each variable influencing surgical delay only after the matching process had been verified as being successful in balancing covariates between the two groups.

**Table 1:** Comparison of demographic data between subjects who did and those who did not have surgery within 36 h of admission

Variables	Time to surgery <36 h	Time to surgery >36 h	*p* value
Number	537 (39.5 %)	824 (60.5 %)	0.01
Age in years	84 (24–103)	83 (31–104)	0.279
Gender	–	–	0.674
Male	143 (26.6 %)	228 (27.6 %)	–
Female	394 (73.4 %)	596 (72.4 %)	–
Fracture type	–	–	0.228
Intracapsular undisplaced	197 (36.7 %)	263 (31.9 %)	–
Intracapsular displaced	156 (29.1 %)	277 (33.6 %)	–
Intertrochanteric	140 (26.1 %)	217 (26.3 %)	–
Subtrochanteric	44 (8.2 %)	67 (8.1 %)	–
Admission source	–	–	0.031^a^
Own home	390 (72.6 %)	650 (78.8 %)	–
Residential/nursing home	118 (22.0 %)	128 (15.5 %)	–
Already inpatient	11 (2.0 %)	24 (2.9 %)	–
Other hospital	2 (0.4 %)	2 (0.2 %)	–
Unknown/other	16 (3.0 %)	20 (2.4 %)	–
Pre-injury walking ability	–	–	0.664
Independent	285 (55.1 %)	439 (53.2 %)	–
1 stick	122 (22.7 %)	203 (24.6 %)	–
2 sticks or frame	99 (18.4 %)	148 (17.9 %)	–
Wheelchair/scooter	12 (2.2 %)	13 (1.6 %)	–
Unknown	19(3.5 %)	21(%)	–

Results are displayed as median (range) for continuous data, and as *n* (%) of population for discrete data

Continuous data were analysed using an independent *t*-test, categorical data using chi-square/Fisher’s test and ordinal data using the Mann–Whitney–Wilcoxon test

^a^Included in the multivariate analysis

**Table 2:** Comparison of clinical data between subjects who did and those who did not have surgery within 36 h of hospital admission

Variable	Time to surgery <36 h	Time to surgery >36 h	*p* value
Co-morbidities			
Dementia	46 (8.6 %)	52 (6.3 %)	0.116^a^
Hypertension	18 (3.4 %)	25 (3.0 %)	0.743
Diabetes mellitus	50 (9.3 %)	89 (10.8 %)	0.375
Ischaemic heart dis.	161 (30 %)	195 (23.6 %)	0.010^a^
COPD/asthma	75 (14 %)	126 (15.3 %)	0.501
Neurological dis.	12 (2.2 %)	17 (2.1 %)	0.830
Stroke	25 (4.7 %)	24 (2.9 %)	0.092^a^
Thyroid dis.	33 (6.1 %)	43 (5.2 %)	0.467
Malignancy	50 (9.3 %)	79 (9.6 %)	0.865
Alcoholism	18 (3.4 %)	25 (3.0 %)	0.743
Chest infection	66 (12.3 %)	97 (11.8 %)	0.773
Urinary tract infection	99 (18.4 %)	123 (14.9 %)	0.087^a^
Myocardial infarction	22 (4.1 %)	17 (2.1 %)	0.028^a^
Cardiac failure	16 (3.0 %)	23 (2.8 %)	0.839
Peripheral vascular dis.	6 (1.1 %)	13 (1.6 %)	0.638^b^
Peptic ulcer dis.	4 (0.7 %)	5 (0.6 %)	0.745^b^
Liver disease	4 (0.7 %)	3 (0.4 %)	0.444^b^
Connective tissue dis.	0 (0 %)	1 (0.1 %)	1.0^b^
Leukaemia	1 (0.2 %)	3 (0.4 %)	1.0^b^
Anaemia	64 (11.9 %)	105 (12.7 %)	0.652
Chronic renal failure	41 (7.6 %)	79 (9.6 %)	0.214
Hyponatraemia	96 (17.9 %)	233 (28.2 %)	0.000^a^
Anticoagulation therapy	7 (1.3 %)	18 (2.2 %)	0.303^b^
Blood results on admission			
HB	12.1 (6–17)	12.0 (7–19)	0.563
Platelet count	264 (43–843)	264 (43–938)	0.313
White cell count	10.3 (4–78)	10.3 (1–67)	0.754
Urea	7.4 (1–34)	7.2 (1–36)	0.950
Creatinine	93 (50–817)	92 (42–512)	0.949
Potassium (K+)	4.4 (2–7)	4.3 (3–7)	0.805
INR	1 (0.8–5.6)	1 (0.8–6.3)	0.540
APTT	29 (20–190)	29 (19–195)	0.450
ASA			0.685
1	52 (9.7 %)	68 (8.3 %)	
2	135 (25.1 %)	226 (27.4 %)	
3	282 (52.5 %)	420 (51.0 %)	
4	68 (12.7 %)	109 (13.2 %)	
5	0 (0 %)	1 (0.1 %)	
Charlson score (median, range)	4 (0–8)	4 (0–9)	0.835

Results are displayed as median (range) for continuous data, and as *n* (%) of population for discrete data

Continuous data were analysed using an independent *t*-test, categorical data using chi-square/Fisher’s test and ordinal data using the Mann–Whitney–Wilcoxon test

Displayed blood results are serum values in mmol/l

*INR* international normalised ratio, *APTT* activated partial thromboplastin time

^a^Included in multivariate analysis

^b^Fisher’s exact test used

## Results

A total 1361 patients underwent hip fracture surgery, of which 537 patients (39.5 %) received surgery within 36 h of admission. The overall median time to surgery from presentation was 23 h (3–36) in group 1 and 72 h (36–774) in group 2. The demographics were similar between patients who did (group 1) and those who did not (group 2) receive timely surgery (Table 1). There was no difference between the two groups with respect to age, gender, walking ability, fracture pattern and ASA grade. However, with regards to admission source, there was a higher proportion of patients presenting from a community care institution in group 1, whilst a higher proportion of patients were from their own home in group 2 (*p* = 0.013).

The distribution of the different co-morbidities between the two groups are summarised in Table 2. There was a higher proportion of patients with cardiac co-morbidities in group 1, while a higher proportion of patients in group 2 presented with hyponatraemia (sodium <135 mmol/l) (*p* = 0.00). There was no difference between the two groups with respect to a number of factors, including pre-existing anticoagulation therapy (*p* = 0.303). Furthermore, the calculated Charlson’s co-morbidity index was also similar between the two groups (*p* = 0.835). There was no statistical difference in haematological and serum biochemical parameters between the two groups (Table 2).

Following univariate analysis, seven variables, including admission source, history of dementia, ischaemic heart disease, MI, cerebrovascular accidents (CVA), urinary tract infections and hyponatraemia met criteria for inclusion into the Cox regression model. The model thereafter inferred only hyponatraemia to be a significant risk factor for delay to surgery beyond 36 h with a covariate adjusted relative risk (RR) 1.24 (95 % CI 1.06–1.44, *p* = 0.006).

The overall 30-day mortality in our cohort of hip fracture patients was 9.0 %. The commonest cause of death was from pneumonia (37 %). Following propensity score analysis, 256 patients with hyponatraemia were matched to 256 patients with normal sodium levels. The absolute acceptable propensity score caliper width was 0.01. A near perfect standardised mean difference of 0.0003 and a variance ratio of 1 (0.01:0.01) confirmed between-group homogeneity to be well within acceptable limits [16]. Thus, the matching process controlled for all collected variables, including time to surgery. The 30-day mortality rates for hyponatraemic patients was 10 % (24/256) and 9 % (22/256) for normonatraemic patients. This was not statistically significant (*p* = 0.757).

## Discussion

Our study has shown that 60.5 % of patients had surgery delayed beyond 36 h. Furthermore, hyponatraemia was identified as a pre-operative risk factor for this. Interestingly, the impact of hyponatraemia on 30-day mortality was not significant.

Nationally, the reason for 37.9 % of patients failing to meet the UK hip fracture BPT target was because of a perceived need for medical optimisation [17]. At first glance the rates of delay in our study may seem high. This was because data collection predated the NICE guidelines for time to surgery. We realised that the guidelines could have modified clinical practice owing to the need for expediting surgery within 36 h. Hence, potential medical causes for delay that would have otherwise been apparent prior to the guidelines would potentially be missed following its introduction. Thus, pre-guideline data were used in an effort to prevent this potential bias.

The median age in both groups of our cohort was above 80 years with a higher proportion of females. This is in agreement with demographic information published by the NHFD [18]. With respect to admission source, we found that a comparatively higher proportion of patients admitted from their own home with a hip fracture were delayed beyond 36 h. Conversely, a higher proportion of patients admitted from a community care institution were seen in the timely surgery group. It is entirely possible that clinical practice may have inherently favoured expedited treatment owing to fears of complications of delayed surgery in patients who were perceived to be frailer. Such patients are more likely to present from a community care institution than their own home [19, 20]. This is also reflected by the comparatively higher proportion of cardiac related co-morbidities in the early surgery group.

This study found that the mean international normalised ratio (INR) and ranges between the two groups were similar. This is because patients with comparatively higher INRs in group 1 had been aggressively treated to correct the values within the 36-h time frame by using, according to our institution’s formalised protocol, vitamin k therapy. This practice is supported by Gleeson et al. who demonstrated in their cohort of 1080 patients, that an active management strategy for the reversal of warfarin anticoagulation facilitated earlier surgery without increasing complications of thromboembolic events, mortality or 30-day re-admission [21]. Equally, it follows that patients in group 2 with comparatively normal INRs were delayed for other reasons.

We found hyponatraemia to be comparatively more common in the delayed surgery cohort. While we did not formally explore the underlying reasons for this, anecdotally we believe that hyponatraemia was perceived to be associated with peri-operative mortality and morbidity. The association between hyponatraemia and mortality has been demonstrated previously [22]. However, it has also been suggested that severe underlying disease is the cause of death while hyponatraemia is merely another complication of this underlying disease. Hence, while it shows an association, it does not necessarily prove causality. Chawla et al. in their study of just over 45,000 patients found that mortality rates tended to increase as sodium levels changed from normal to mild hyponatraemia. Surprisingly, as hyponatraemia became more severe (sodium <120 mmol/l) mortality trends reversed [23]. Furthermore, over the 12 years of their study, only three deaths were directly attributable to adverse hyponatraemia sequelae. Our study also found no difference in mortality incidence between hypo- and normonatraemic patients after matching groups for confounders, such as liver and renal failure, which may have contributed to both mortality and hyponatraemia. This supports the notion that hyponatraemia may not necessarily be singularly causal to mortality. Subgroup analysis of those with severe hyponatraemia (sodium <120 mmol/l) was unfortunately precluded because there were only three patients that fell into this category. We cannot therefore draw conclusions as to whether severe hyponatraemia is a risk factor to mortality and whether it is reasonable to delay surgery in its presence.

Interestingly, we found that patients with a history of ischaemic heart disease were significantly less likely to have their surgery delayed beyond 36 h (23.6 versus 30 %, *p* = 0.01). Patients with a history of myocardial infarction also were significantly less likely to have delayed surgery (4.1 versus 2.1 %, *p* = 0.03). These patients may have been prioritised as these risk factors are non-modifiable and clinical opinion may have been not to increase their risk further by also having delayed surgery. Similarly, there is an increased awareness of the need to avoid unnecessary delays in order to gain financial compensation for services used in treating such patients, and to avoid increased costs associated with longer hospital stays in these patients [24, 25].

The main weakness of this study lies in the fact that we present data pertaining to only one major trauma unit. One may argue that patient data from other units may yield differing results. However, our findings may be more widely generalisable as our patient population demographics and mortality rates of 9.0 % (*n* = 123/1361) at 30 days were comparable to other published studies and NHFD reports [18, 26, 27]. Although retrospective by design, we cross-referenced prospectively collected data from multiple sources, including a national hip fracture registry and our own hospital-coding database, ensuring that the final dataset was reliable. Non-medical risk factors for delay are not available in the NHFD or medical notes and hence our regression model is limited by their absence. We have, however, made a comprehensive assessment of 38 medical and demographic variables. These variables are readily available on initial presentation and are thus easily collectable by other units who also wish to make similar assessments of their services.

This type of study is relevant in the current NHS culture of target-driven quality health care delivery. Verifying and investigating the legitimacy of medical causes of surgical delay is therefore not only pertinent, but has also been specifically highlighted as a vital area for future research by the NHFD Scientific Committee [9]. To our knowledge this study is unique in assessing the risk factors to delay in achieving the 36-h BPT target in these patients. Nationally, delays are also due to a lack of theatre time, equipment or high dependency beds (43 % of the time) [17]. Therefore, whilst streamlining medical decision-making may help improve the likelihood of attaining the BPT, availability of clinical resources plays an important part.

In conclusion, surgical delays can result when one aims to avoid medical complications associated with hastened hip fracture surgery. However, delay is not justifiable in the presence of non-severe and isolated hyponatraemia. Instead, surgical delay should only be warranted in the presence of medical conditions which contribute to mortality and are optimisable.
